# Interstate Variation in the Burden of Fragility Fractures

**DOI:** 10.1359/jbmr.081226

**Published:** 2008-12-08

**Authors:** Alison B King, Anna NA Tosteson, John B Wong, Daniel H Solomon, Russel T Burge, Bess Dawson-Hughes

**Affiliations:** 1 Public Policy and Government Relations, Procter & Gamble Health CareNorwich, New York, USA; 2 The Dartmouth Institute for Health Policy and Clinical Practice, Dartmouth College and the Multidisciplinary Clinical Research Center in Musculoskeletal Diseases, Dartmouth Medical SchoolLebanon, New Hampshire, USA; 3 Department of Medicine, Division of Clinical Decision Making, Tufts Medical CenterBoston, Massachusetts, USA; 4 Division of Rheumatology, Brigham and Women's HospitalBoston, Massachusetts, USA; 5 Global Health Economics and Outcomes Research, Abbott Laboratories, Abbott ParkIllinois, USA; 6 College of Pharmacy, University of CincinnatiCincinnati, Ohio, USA; 7 Jean Mayer USDA Human Nutrition Research Center on Aging, Tufts UniversityBoston, Massachusetts, USA

**Keywords:** osteoporosis, economic analysis, epidemiology, geography of health, race and ethnicity

## Abstract

Demographic differences may produce interstate variation in the burden of osteoporosis. We estimated the burden of fragility fractures by race/ethnicity, age, sex, and service site across five diverse and populous states. State inpatient databases for 2000 were used to describe hospital fracture admissions, and a Markov decision model was used to estimate annual fracture incidence and cost for populations ≥50 yr of age for 2005–2025 in Arizona (AZ), California (CA), Florida (FL), Massachusetts (MA), and New York (NY). In 2000, mean hospital charges for incident fractures varied 1.7-fold across states. For hip fracture, mean charges ranged from $16,700 (MA) to $29,500 (CA), length of stay from 5.3 (AZ) to 8.9 days (NY), and discharge rate to long-term care from 43% (NY) to 71% (CA). In 2005, projected fracture incidence rates ranged from 199 (CA) to 266 (MA) per 10,000. Total cost ranged from $270 million (AZ) to $1,434 million (CA). Men accounted for 26–30% of costs. Across states, hip fractures constituted on average 77% of costs; “other” fractures (e.g., leg, arm), 10%; pelvic, 6%; vertebral, 5%; and wrist, 2%. By 2025, Hispanics are projected to represent 20% of fractures in AZ and CA and Asian/Other populations to represent 27% of fractures in NY. In conclusion, state initiatives to prevent fractures should include nonwhite populations and men, as well as white women, and should address fractures at all skeletal sites. Interstate variation in service utilization merits further evaluation to determine efficient and effective disease management strategies.

## INTRODUCTION

Aging of the U.S. population will increase the societal burden of diseases such as osteoporosis that disproportionately affect the elderly. The prevalence of osteoporosis and low bone mass in the United States is expected to increase from 43.6 million in 2002 to 61.4 million in 2020.(1) The burden on the U.S. healthcare system was estimated to be $19 billion in direct cost of osteoporosis-related fractures in 2005, including $17 billion for incident fractures and $2 billion for ongoing costs of prevalent hip, vertebral, and pelvic fractures.(2) If current medical practice patterns continue, the direct medical cost of osteoporosis is projected to increase by nearly 50% from 2005 to 2025, reaching $25 billion for incident fractures.(2) These estimates reflect the marked demographic changes expected over the next two decades. The U.S. population ≥65 yr of age is predicted to increase 104% from 2000 to 2030, reaching 71.4 million.(3) The largest increases are expected in nonwhite populations.

Although U.S. incidence of hip fractures began declining in 1997,(4) possibly as a result of improvements in the prevention and treatment of osteoporosis, recent research suggests that this progress may be inconsistent across sexes and ethnic groups.(4,5) Assessment of hip fracture incidence in California from 1983 to 2000 showed that the incidence of hip fractures declined or remained unchanged among non-Hispanic women and black or Asian women and men, yet increased in Hispanic women and men.(5) Furthermore, there is evidence that diagnosis and treatment are inadequate in nonwhite women and men,(6) that blacks receive less osteoporosis-related healthcare than whites,(7) and that service use differs among ethnic groups after fracture.(8) Mortality after hip fracture is higher in men and black women than in white women.(9,10) Research on the current and future burden of osteoporosis on the U.S. healthcare system projects that fracture incidence will increase nearly 175% in Hispanic and other nonwhite populations over the next two decades.(2) Demographic differences across states may therefore result in significant differences in the burden of osteoporosis at the state level.

Understanding interstate variation in osteoporosis incidence, healthcare delivery, and fracture costs may help government and private healthcare programs plan for future resource needs, as well as target interventions to prevent or manage the disease. However, to date, little is known about the cost of osteoporosis at the state level. Several reports have estimated state-level hospital costs associated with osteoporosis,(11,12) the overall burden of osteoporosis,(13,14) or future impact of demographic changes.(14) However, differences in methodology between studies have precluded interstate comparisons. This study was undertaken to describe hospital care patterns and to estimate the incidence and costs of fragility fractures by race/ethnicity, age, sex, fracture type, and health service site in five states.

## MATERIALS AND METHODS

### Overview

Using state inpatient databases for 2000, we conducted a descriptive analysis of hospital admissions for fragility (low-impact or nontraumatic) fractures in women and men ≥50 yr of age for the states of Arizona, California, Florida, Massachusetts, and New York. We then used a Markov model to estimate total fracture incidence and associated direct medical costs for 2005. These estimates were based on state-specific hospital data and published data on fracture incidence and cost in the long-term care and outpatient settings, updated with demographic projections and medical care price indexes. Population data were used to project annual costs for 2005 through 2025. We included California, Florida, and New York because they have the highest populations of Medicare beneficiaries.(15) We included Arizona because earlier studies of 16 states showed Arizona to have the shortest hospital length of stay (LOS) (Procter & Gamble Pharmaceuticals, data on file, 2002–2003) and because high growth of the senior population is projected.(16) We included Massachusetts because it has a large number of academic medical centers and high per capita healthcare spending,(17,18) and it is the most populous New England state.(19) State hospital data for 2000 and total fracture incidence and cost for 2005 are reported here. For comparison, we included national hospital data for 2001(20) used in a previous analysis, along with national fracture incidence and costs for 2005.(2)

Osteoporosis-related fractures included fractures of the hip, vertebrae, and wrist/forearm, as well as pelvic and “other” (nonpathologic humerus, clavicle/scapula/sternum, femur, hands/fingers, patella, tibia, fibula) fractures.

### Hospital data

Each state's inpatient database for 2000(21) was used to determine LOS, primary payer, and discharges to long-term care (LTC) facilities by fracture type, patient age, ethnicity/race, and sex.

In the descriptive analysis of state hospital admissions and subsequent modeling, hospital cases included closed fractures with ICD-9-CM primary diagnosis codes of 820.0x, 820.2x, and 820.8x for hip fractures; 805.0x, 805.2x, 805.4x, and 805.8x for vertebral fractures; 813.2x, 813.4x, and 813.8x for wrist/forearm fractures; 808.0x, 808.2x, 808.4x, and 808.8x for pelvic fractures; and 810.0x (clavicle); 811.0x (scapula); 812.0x, 812.2x, 812.4x (humerus); 814.0x (carpal bones); 815.0x (metacarpal bones); 821.0x (unspecified parts of femur); 822.0x (patella); and 823.0x, 823.2x, 823.4x, and 823.8x (tibia and fibula) for “other” fractures. Hospital admissions for fractures caused by severe trauma were excluded; such fractures were identified with the following E-codes: E800–E848 for transportation accidents; E916–E923 for struck, objects, machines, instruments, firearms, and explosions; E928.8–E928.9 for other and unspecified; E950–E978 for suicide, homicide, and legal intervention; E988 for undetermined injury; and E999 for war. In addition, the analysis excluded vertebral fractures with neoplasm as a secondary diagnosis. High-charge outlier trims were imposed, based roughly on the 99th percentile cost distribution for each fracture type, to better ensure exclusion of extreme costs not likely related to fracture events. Thus, charges were excluded if greater than or equal to $100,000 for hip and pelvic, $50,000 for “other,” and $30,000 for vertebral and wrist fractures.

### Population data

Population data were obtained from the U.S. Census Bureau.(16) Categorization of race/ethnicity differed slightly across geographic areas, primarily because of differences in reporting of nonwhite Hispanics. To match the race/ethnicity categories used in the hospital discharge and population data, we collapsed ethnic groups into the following categories: White, Black, Hispanic, and Other. White includes only non-Hispanic whites; Black includes non-Hispanic blacks; Hispanic includes Hispanic of any race; and Other includes American Indian, Eskimo, Aleut, Asian/Pacific Islander, and others of non-Hispanic origin.

### Model

A Markov state-transition model of osteoporosis was used to estimate the total number of fracture events and related costs in women and men 50–99 yr of age in the base year 2005 and subsequent years.(22) The model follows patient cohorts over time from a healthy state through future health states, such as bone fracture and recovery. The probabilities of transition between health states were derived from state-specific mortality data,(23,24) state hospital data on hip fracture incidence,(21) and a published epidemiologic study by Melton et al.(25) on the incidence of nonhip fractures, many of which are managed in the outpatient setting. In their study, Melton et al. included only new (incident) fractures that came to clinical attention, and vertebral fractures were confirmed radiologically. To estimate nonhip fractures in nonwhite populations, we multiplied these published nonhip incidence rates by the ratio of each race's hip fracture incidence rates (by age and sex) to whites' hip fracture rates in each state. Annual fracture incidence was estimated from 2005 to 2025 on the basis of demographic projections.(16) The model and study methodology have been described in detail elsewhere.(2,22)

### Costs

Total medical costs were estimated from state-specific hospital inpatient charges,(21) discharge rates to LTC,(21) and per diem costs in LTC facilities,(26–29) along with published national outpatient, inpatient physician, rehabilitation, and short-stay hospitalization costs(30,31); LTC treatment pathways(32); and ratios of outpatient or LTC to inpatient care costs.(33) Unit costs for each fracture type were estimated by age, sex, and race/ethnicity, as shown for U.S. white women in Appendix 1 of Burge et al.(2) For hospital inpatient care, mean inpatient facility charges were calculated after exclusion of trauma-related cases, pathological fractures, cases with secondary diagnosis of fracture, and high-cost outliers. Hospital charges were converted to costs using a national cost-to-charge ratio of 0.61.(34) Because race-specific data were available only for hospital inpatient care, outpatient costs were estimated by multiplying the state-specific unit cost for inpatient care (by age, sex, and race/ethnicity) by the national ratio of outpatient physician, hospital, or other cost to inpatient cost for each fracture type.(33) Unit costs were converted to 2005 terms on the basis of corresponding medical care price indexes. Costs were calculated annually through 2025 according to demographic projections, which adjusted for differential growth in all population subgroups, including those within the Other category. Medical costs and practices were assumed to remain constant.

### Sensitivity analyses

In one sensitivity analysis, we modeled ongoing costs from prevalent hip, spine, and pelvic fractures that occurred during the previous 5 yr according to the methodology described by Burge et al.(14) In other analyses, we assessed the sensitivity of the results to ±25% variation in unit costs, along with a cost-to-charge ratio of 0.5 for the low-cost scenario and ±10% variation in fracture incidence rates.

## RESULTS

### Hospital admissions and costs, 2000

**FIG. 1:**
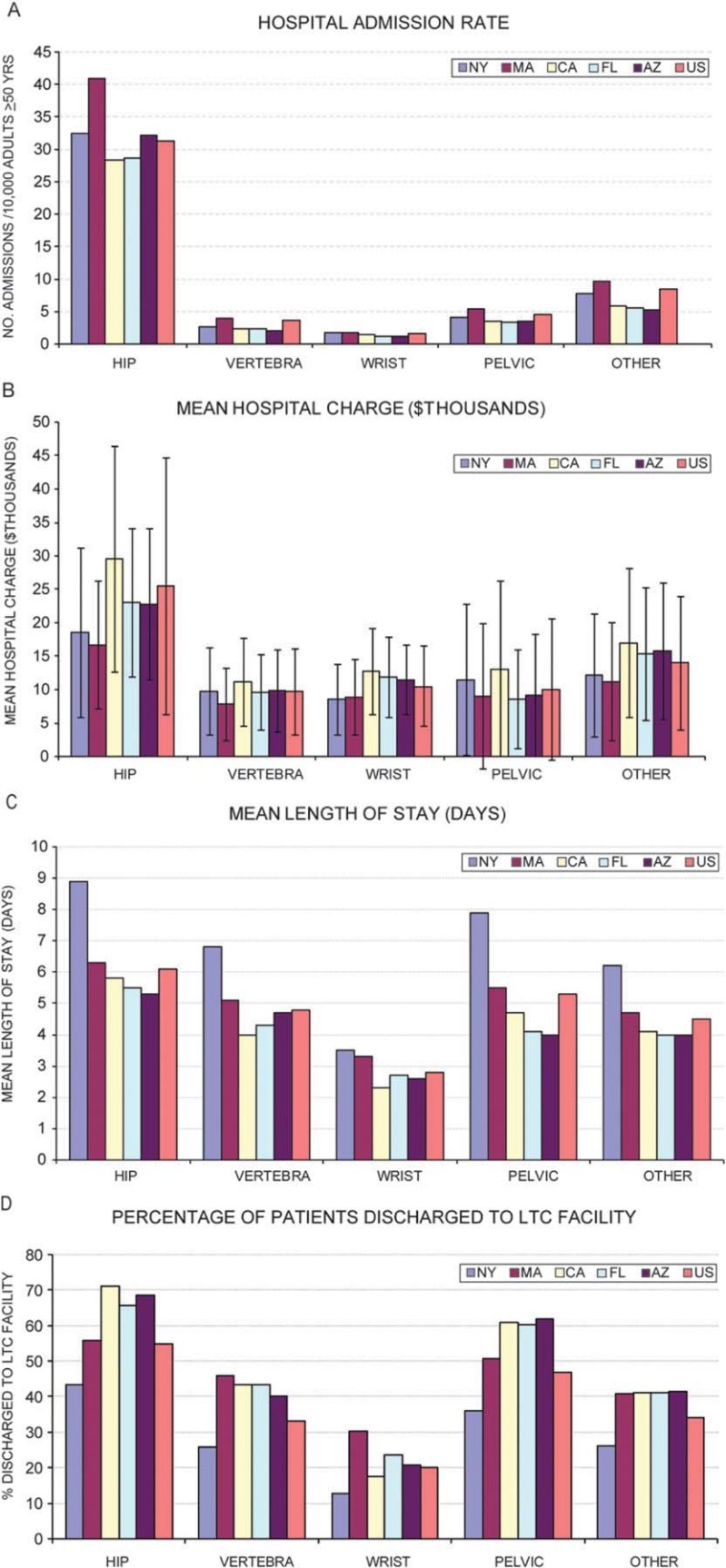
(A) Number of hospital admissions per 10,000 men and women ≥50 yr of age. (B) Mean hospital charge (bars represent SDs). (C) Mean length of hospital stay. (D) Percentage of patients discharged to long-term care (LTC) facilities for men and women ≥50 yr of age admitted to hospitals for fragility fracture in 2000. (U.S data are for hospital admissions in 2001.)

In all five states, hospital admission rates per 10,000 were highest for hip fractures in 2000 (28.3–40.9), followed by “other” (5.3–9.6), pelvic (3.4–5.4), vertebral (2.1–3.9), and wrist fractures (1.1–1.8) (Fig. 1A); thus, hip fractures constituted the majority of fracture admissions (66–73%), followed by “other” (12–16%), pelvic (8–9%), vertebral (5–6%), and wrist (3–4%) fractures. In 2000, mean hospital charges for all incident fractures varied 1.7-fold across states. For hip fracture, the mean charge ranged from $16,700 in Massachusetts to $29,500 in California, compared with a U.S. average of $25,500 in 2001 (Fig. 1B). The interstate differences were not explained by LOS. For hip, pelvic, and “other” fractures, LOS was longest in New York, followed by Massachusetts, California, Florida, and Arizona (Fig. 1C). Discharge rates to LTC facilities after these fractures tended to be inversely related to LOS, with the lowest rate in New York, followed by Massachusetts (Fig. 1D).

### Predicted total fractures and medical costs, 2005

The predicted incidence of fragility fractures and total direct medical cost in 2005 for women and men 50–99 yr of age are shown in Tables 1 and 2. Hospital inpatient costs represented roughly one half of total costs, ranging from 45% in Massachusetts to 56% in California, and LTC represented one third or more of total costs, ranging from 32% in California to 42% in Massachusetts. The per capita incidence and cost of fractures were highest in Massachusetts. The per capita fracture incidence was lowest in California, whereas the per capita cost was lowest in Florida and New York. Per capita incidence and cost by sex, race/ethnicity, and fracture type are shown in Appendixes 1 and 2.

**Table 1:** Estimated Incident Fractures in Five States and the United States for 2005 for Population ≥50 yr of Age

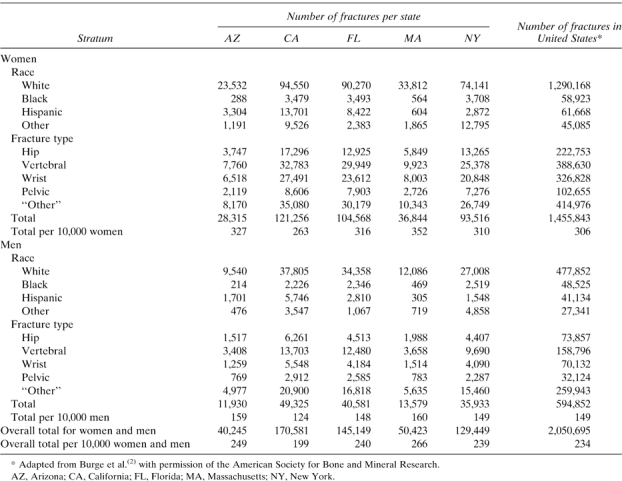

**Table 2:** Estimated Fracture Costs^*^ in Five States and the United States for 2005 for Population ≥50 yr of Age

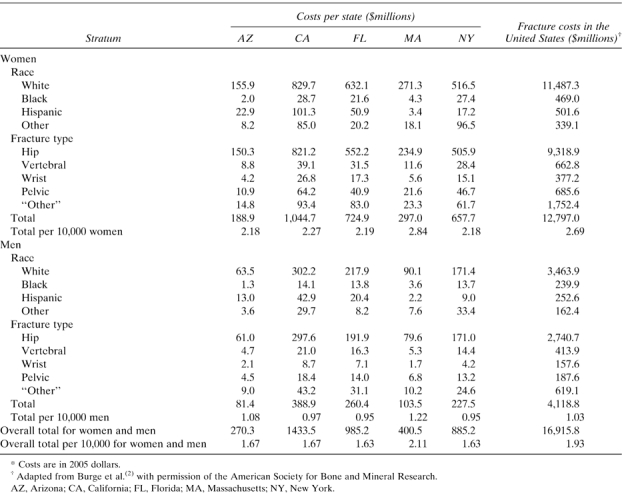

The distributions of fractures and costs by fracture type were similar across states (Tables 1 and 2). The aggregate “other” fracture type was the most common in each state, accounting for 28% or 29% of the fractures in women and 41–44% of fractures in men. Across the five states, hip fractures generated 77% of costs; “other” fractures, 10%; pelvic fractures, 6%; vertebral fractures, 5%; and wrist fractures, 2%.

The majority of fractures (71–75%) occurred in the Medicare population ≥65 yr of age, representing 88–89% of each state's costs (data not shown). Overall, men accounted for 27–30% of fractures and 26–30% of costs in the five states. For both sexes, the distribution of fracture costs by age reflected the higher incidence of lower-cost wrist and “other” fractures in younger age groups and the increasing proportion of higher-cost hip and pelvic fractures in older age groups. However, the proportion of fractures occurring in the youngest age group (50–64 yr) was higher for men (31–39%) than women (23–26%). In all states, fracture costs jumped substantially in the two older age groups, and costs were 3- to 4-fold higher in the oldest (≥85 yr) than the youngest group (50–64 yr). Fracture costs among the oldest patients (≥85 yr) ranged from $97.5 million in Arizona to $556.6 million in California.

**FIG. 2:**
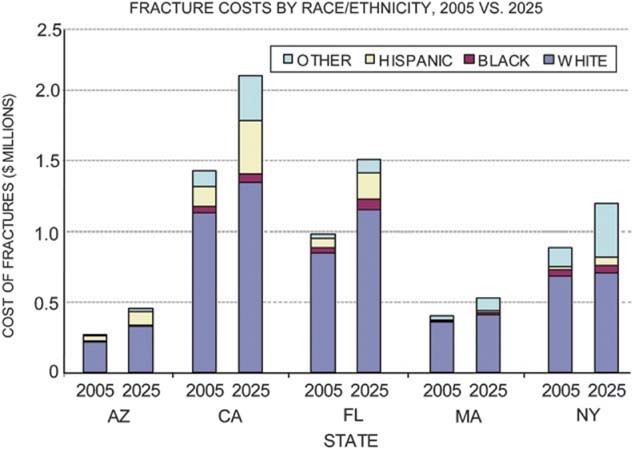
Distribution of estimated fracture costs by race/ethnicity in 2005 and 2025, by state.

The distribution of fractures by race/ethnicity and corresponding costs (Fig. 2) varied across the five states. Hispanics represented a smaller proportion of fractures in Massachusetts (1.8%) and New York (3.4%) than in Arizona (12.4%), California (11.4%), and Florida (7.7%). The Other race/ethnicity group accounted for 13.6% of fractures in New York, 7.7% in California, 5.1% in Massachusetts, 4.1% in Arizona, and 2.4% of fractures in Florida. Asians/Pacific Islanders constituted nearly all (94–95%) of the Other category in three states (California, Massachusetts, and New York), 82% of women and 85% of men in Florida, 41% of women and 42% of men in Arizona, and 66% of women and 71% of men in the United States.

### Projected total fractures and medical cost, 2005–2025

Projected fracture incidence in 2025 ranged from 67,000 in Arizona to 254,000 in California. Growth in fracture incidence from 2005 to 2025 is projected to be lowest in Massachusetts and New York (31%), followed by California (49%), Florida (57%), and Arizona (67%), with costs following a similar pattern (Table 3). Growth in fracture incidence and cost varied across age, sex, and ethnic groups. The increases for men outpaced those for women; in 2025, fractures in men represented 29–31% of each state's fractures, and costs reached 28% (New York) to 33% (Arizona) of total costs. In all five states, the largest increase in fractures was projected for the 65- to 74-yr-old group; increases of 100% or more were projected for Arizona, California, and Florida (data not presented). By 2025, Hispanics were projected to account for one fifth of fractures in Arizona and California. The Other race/ethnicity population was projected to account for 27% of fractures in New York, 13% in California, and 12% in Massachusetts. Fracture costs among Hispanics more than doubled in all five states, with >3-fold growth in Massachusetts, where Hispanics represented a small proportion of fractures in 2005 (Fig. 2). In all states except Arizona, fracture costs increased roughly 3-fold among the Other population. By 2025, Asians/Pacific Islanders are projected to constitute 96–97% of women and men in the Other category in three states (California, Massachusetts, and New York); 85% and 87% in Florida, respectively; 50% in Arizona; and 72% in the United States.

**Table 3:** Projected Direct Medical Cost of Fractures in Five States and the United States From 2005 Through 2025

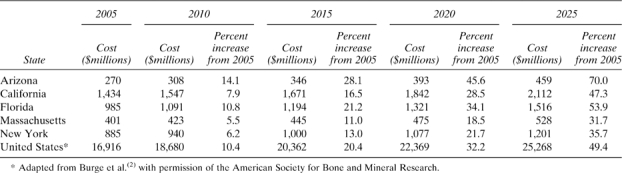

### Sensitivity analyses

Including current-year (2005) costs of prevalent hip, pelvic, and vertebral fractures that occurred during 2000–2004 increased base year (2005) cost by 18% in Arizona, 17% in Massachusetts, 15% in Florida and New York, and 14% in California, compared with 14% for the United States. When fracture incidence rates and unit costs were altered, sensitivity analyses showed similar effects across the five states and the United States. Among fracture types, hip fracture incidence had the largest impact on total cost, followed by “other” fractures, as previously reported.(2)

## DISCUSSION

This study compares the burden of osteoporosis across five state populations using a validated model to estimate fracture incidence and costs by race/ethnicity, sex, and fracture type. The distribution of fracture types was consistent across states, whereas significant differences were seen among states in per capita fracture incidence and cost, hospital care patterns, and the distribution of fractures across race/ethnicity groups.

Geographical differences in healthcare spending and medical care patterns are well documented.(35–37) Our study provides evidence of regional variation in healthcare efficiency for patients with fragility fractures. Per capita medical spending on fragility fractures was similar in Arizona, California, Florida, and New York ($163–$167), despite a range in per capita fracture incidence from 199 to 249 per 10,000. Both per capita medical cost ($211) and fracture incidence (266 per 10,000) were highest in Massachusetts, which is known for high per capita healthcare spending. Although Massachusetts' high spending has been attributed to intensive use of hospital services and its high concentration of teaching hospitals,(18,38) the mean LOS in Massachusetts was similar to the U.S. average, and Massachusetts had the lowest mean hospital charge for admissions with a primary diagnosis of hip fracture. Thus, fracture incidence and nonhospital care may be cost drivers in Massachusetts. In New York, mean LOS was far greater than in the other four states, whereas average hospital charge was not. In states where patients had shorter LOS after hip or pelvic fracture (Arizona, California, Florida), larger proportions were discharged to LTC facilities. These data have near- and long-term implications for health service allocation, such as availability of LTC beds and rehabilitation facilities. Premature hospital discharge may increase risk of adverse clinical outcome. A prospective study of hip fracture patients admitted to metropolitan New York hospitals in 1997 and 1998 found that patients with active clinical issues or new impairments on discharge had increased risk-adjusted rates of death or readmission.(39) Further study is needed to understand whether interstate differences in hospital care after fracture affect the quality of care and patient outcomes.

One limitation of our cost estimates is the lack of adjustment for differences in patient case-mix. Data on patient comorbidities became available in the state HCUP databases beginning in 2005. Research suggests, however, that patient characteristics, such as age and comorbidities, would not explain the substantial interstate differences in hospital care patterns and efficiency of medical care. Fisher et al.(40) found that for patients with hip fracture, higher regional Medicare spending correlated with receiving more services but not with better health outcomes, after controlling for regional variation in illness and price.

Demographic differences among states were reflected in the distribution of fractures across race/ethnicity groups in 2005 and in shifts in the predicted fracture burden over the following two decades. Substantial growth in fractures and costs is projected in all five states by 2025, with fracture growth in Arizona roughly twice the rate for Massachusetts and New York. The nonwhite population is predicted to incur a larger share of the disease burden in each state, particularly the Hispanic populations in Arizona, California, and Florida and Other populations in New York, California, and Massachusetts. The latter reflects growing Asian populations. Asian/Pacific Islanders are projected to constitute nearly all (96–97%) of the Other population in these three states and only half of the Other population in Arizona, the other half being American Indian/Aleutian/Eskimo. Men are predicted to incur an increasing proportion of fracture costs as a result of demographic shifts alone, reaching one third of costs in Arizona in 2025. Thus, our research indicates the continued need for education and interventions to overcome the perception of osteoporosis as a white woman's disease.

This study also highlights the impact of fractures at skeletal sites beyond the spine, hip, and wrist. As in our earlier study of U.S. fracture burden,(2) we found that ∼40% of fractures in each state occurred at the pelvis (7%) and “other” (32–33%) skeletal sites such as leg, arm, and shoulder. The high rate of “other” fractures in younger (age, 50–64 yr) men compared with younger women in our study suggests the need for further evaluation of fractures in men to understand which of these are fragility fractures. Exclusion of E codes E880–E883 (falls involving ladders, stairs, holes, buildings), in addition to codes for major trauma, may be appropriate in future analyses. Nonetheless, our cost estimates for “other” fractures seem more conservative than costs reported earlier by Ray et al.(33) Pelvic and “other” fractures combined accounted for a lower proportion of U.S. (19%) and state costs (15–20%) than the 29% reported by Ray et al. for the United States in 1995. This difference may be explained at least in part by methodology rather than changes in fracture incidence. Our study included only patients with a primary diagnosis of fracture, whereas Ray et al. included hospital patients with a secondary diagnosis of fracture, estimating excess hospital days attributable to osteoporosis for these patients. They found fractures at sites other than the traditional hip, spine, and forearm/wrist to constitute 33% of hospital discharges with a primary diagnosis of fracture but 62% of discharges with a secondary diagnosis of fracture. Thus, Ray et al. found pelvic and “other” fractures to represent 26% of hospital costs for fragility fracture compared with our 19% for the United States and 13–18% in five states (data not presented).

In each of the five states and the United States in 2005, the proportions of total estimated costs for hospital care and LTC differed from those estimated for 1995 by Ray et al. based on 1992 hospital data and 1985 LTC facility data.(33) In our study, hospital care for fragility fractures represented a smaller proportion of total estimated costs (45–56%) and LTC a larger proportion (32–42%) than the 62.4% and 28.2%, respectively, estimated by Ray et al.(33) These differences may reflect a shift from hospital inpatient care to nursing home and home healthcare in the 1980s and much of the 1990s.(32,41,42) Our data also differ from the estimate by Max et al.(13) that care in LTC facilities comprised 59% of total costs for osteoporosis in California in 1998. As in our study, their evaluation based LTC admissions on California hospital discharge rates to LTC; their 73% discharge rate after hip fracture in 1998 was identical to the 73% we found in 2000. However, they assumed that only 61% of nursing home patients were admitted from hospitals. Their study also found substantially higher numbers of hospital admissions for osteoporosis and related fractures (4-fold higher for “other” fractures) and lower mean hospital costs for all fracture types except vertebral fractures than our study. These differences may result from our more stringent exclusions and application of different cost-to-charge ratios in the two studies.

Consensus has not been reached on the extent to which morbidity and mortality associated with fractures are attributable to osteoporosis.(43) We found significant inpatient and LTC costs for the small proportion of patients with vertebral or wrist fractures who are hospitalized. Additional studies may elucidate whether these LTC admissions stem from comorbid conditions or perhaps older patients' challenges with activities of daily living. In addition, the contribution of osteoporosis to high-trauma fractures merits study. Mackey et al.(44) recently reported that low BMD was associated with increased age-adjusted risk of both high- and low-trauma fractures in older women and men.

Our estimates used state-specific demographic and hospital data, discharge rates to LTC, and per diem costs in LTC facilities. In-depth analysis of interstate differences is impeded by data limitations, such as lack of all-payer claims data for outpatient and LTC settings and variable composition of state hospital (HCUP) databases. For example, the percentages of community hospitals included in the five state HCUP databases ranged from 85% (Massachusetts) to 100% (New York).(45) Furthermore, noncommunity hospitals constituted 9–10% of the HCUP databases in California and Florida compared with 4% or less in the other three states, and federal hospitals were included in the databases for some (Arizona, California, and Massachusetts) but not all of the five state databases.(46) Like earlier studies, our work is also constrained by its reliance on published data to generate fracture incidence rates and unit costs for the outpatient and LTC settings. In the absence of data, we assumed that outpatient costs represent a uniform proportion of total costs across race/ethnic groups.

Our research suggests that there are geographic differences in fracture burden within and across race/ethnic groups, such as higher fracture rates among Hispanics in Arizona than in New York and higher rates among black men than women in Massachusetts and the United States (Appendix 1). These differences are driven by hip fracture incidence, as per our study methodology. Fang et al.(47) also reported higher hip fracture rates for black men than women in age groups 50–69 yr in New York city during 1988–2002. In Massachusetts and the United States, we found higher rates for black men than women in the oldest age group (age 85+ yr), as well as younger ages. Unlike earlier studies,(5,47) we also found higher fracture rates for the Other population than whites in several states (Florida, Massachusetts, and New York). These differences do not seem to be explained by age adjustment in the earlier studies. To understand and address potential disparities in fracture prevention and care, further state-level research is needed on outpatient care, home healthcare, and length of stay in rehabilitation and skilled- and intermediate-care facilities for various race/ethnicity groups. We relied on a single epidemiology study conducted in predominantly white Olmsted County, MN,(25) to estimate incidence rates for fractures not associated with a hospital admission, and we adjusted incidence rates for nonwhite populations on the basis of race-specific hip fracture incidence rates. This methodology may either under- or overestimate nonhip fracture incidence rates for nonwhites in the five states and the United States. Moreover, the Olmsted study reported pelvic fracture incidence rates six times higher than a Leicestershire, England, study of hospital admissions(48) and 3.6–5.3 times higher than our state hospital admission rates for pelvic fracture. These differences may stem from variation in hospital coding or fracture incidence.

Sensitivity analyses assessed the uncertainty in unit cost assumptions, showing hip fracture to have the greatest impact on total cost. As reported previously,(2) our unit cost estimates for hip fracture were considerably below most previously published estimates when updated to 2005 dollars.(31,33,49) Sensitivity analysis showed a 14–18% increase in 2005 costs in five states and the United States when the model included ongoing costs from prevalent hip and pelvic fractures that occurred in the previous 5 yr. Additionally, our model considered only direct healthcare costs in a given year. Consideration of long-term indirect costs would produce substantially higher estimates of fracture burden. Although fractures occur predominantly among retirees, their caregivers are often working adults. The lifetime attributable cost of hip fracture has been estimated to be $81,300 in 2001, of which $24,600 was informal home care.(32)

Many of our modeling assumptions were based on publications that date from >5 yr ago and may not reflect current modes of healthcare delivery. Our projection methodology also assumed that medical practice, fracture risk, technology, and costs for treating fractures remain unchanged over time, which is unlikely. For example, recent research suggests growing disparities in fracture risk across sex and race/ethnicity. Hospitalization rates for hip fractures decreased among older U.S. women from 1993 to 2003 but increased for men.(4) Similarly, hip fracture incidence among Hispanics in California doubled from 1983 to 2000, whereas incidence declined among white women and remained constant among black and Asian women.(5) Such a trend would magnify the already high projections for fracture growth among Hispanic populations. These data highlight the importance of changing the paradigm for prevention and management of fragility fractures to ensure care addresses local and regional needs and is inclusive of men, women, and ethnic populations. The World Health Organization risk prediction algorithm (i.e., FRAX), which has been tailored to various U.S. racial/ethnic populations, may be helpful for counseling diverse populations.(50)

In conclusion, interstate differences in the burden of fragility fractures reflect differences in state demographics and variation in medical practice and healthcare delivery systems. Our research highlights the need for awareness among patients, clinicians, and caregivers that osteoporosis can cause fractures at multiple skeletal sites, not just those traditionally associated with osteoporosis, and the disease can strike men and nonwhite women. In nearly all of the states studied, fracture incidence is expected to double among Hispanics and triple among the Other/Asian population in just 20 yr. These projections may help states anticipate future demand for healthcare services, such as hospital and nursing home beds, and develop culturally appropriate interventions to forestall expected growth.

## Appendix

**APPENDIX 1:** Estimated Incident Fractures in Five States and the United States for 2005 per 10,000 Population ≥50 Yr of AGe

**APPENDIX 2:** Estimated Fracture Costs^*^ in Five States and the United States for 2005 per 10,000 Population ≥50 Yr of Age
